# The Supplement of Magnesium Element to Inhibit Colorectal Tumor Cells

**DOI:** 10.1007/s12011-022-03393-2

**Published:** 2022-08-25

**Authors:** Heng Li, Xiaonan Feng, Hai Li, Shuo Ma, Wei Song, Bao Yang, Tao Jiang, Chun Yang

**Affiliations:** 1 Department of Colorectal Surgery, General Hospital of Ningxia Medical University, Yinchuan, 750004 China; 2 Department of Nephrology, General Hospital of Ningxia Medical University, Yinchuan, 750004 China

**Keywords:** Magnesium ions, Colorectal tumor, Tumor inhibition, In vitro, In vivo

## Abstract

Magnesium ions are essential elements to the human body, with a daily intake of about 350 mg for an adult. Recently, a meta-analysis reported that magnesium ion intake is related to a reduced risk of colorectal tumors. In addition, implantation of biodegradable magnesium pins after colorectal tumor resection could potentially inhibit the residual tumor cells. These impressive results implied that magnesium ions possess inhibitory properties against colorectal carcinoma. However, this hypothesis has yet to be confirmed by experimental results. In this work, different concentrations of magnesium ions were modulated to investigate their inhibitory effects on cell viability through cell cycle arrest, subsequently inducing apoptosis by activating the caspase-3 pathway. The animal experiments revealed that magnesium injection restricted tumor growth after 3 weeks of treatment compared to the control group. According to the immunohistochemistry and transmission electron microscopy results, the remarkable effect may be attributed to promoting the apoptotic rate of tumor cells. The evidence highlights the potential for the clinical use of magnesium implants to inhibit the growth of residual cells after colorectal tumor surgery.

## Introduction

Colorectal carcinoma (CRC) is the most widespread cancer of the intestinal tract system and is a major public health issue. In 2020, CSC had a 10.0% incidence with a 9.4% mortality rate and was one of the three most common oncological diseases, gradually affecting younger patients [1–3]. Combined surgical removal and chemotherapy is a typical treatment for CRC. However, postoperative recurrence and metastases remain a challenge for surgeons and patients, with the risk of secondary surgery or even life-threatening consequences. Recently, epidemiologic studies have indicated that magnesium (Mg) intake may decrease the risk of CRC, although this observation is not universal [4, 5]. Zan et al. [6] reported that high-purity magnesium staples inhibit the viability and migration of CRC cells mainly due to the released hydrogen inducing tumor cells apoptosis through the p53-lysosome-mitochondria pathway [7]. However, further research is required to investigate the effects of magnesium ions (Mg^2+^) on CRC. Mg^2+^ is the second most plentiful divalent cation in the human body and is involved in hundreds of physiologic processes associated with energy metabolism and nucleic acid synthesis [8]. The “Dietary Reference Intakes (DRIs)” recommends a daily intake of 220–350 mg of Mg^2+^ for adults [9]. As a nutritional element in the body, Mg^2+^ supplementation is beneficial for several medical conditions, such as inflammation [10], bone regeneration [11, 12], arrhythmia [13], and depression [14]. The homeostasis of Mg^2+^ is regulated through the circulatory system and excessive Mg^2+^ can be excreted via the feces and urine [15]. Therefore, adverse effects due to Mg^2+^ accumulation are rare. Recent studies reported that serum Mg^2+^ levels are associated with the immune system, and an Mg^2+^-rich environment promotes CD 8 + T cells to eliminate abnormal or infected cells [16]. Furthermore, Mg^2+^ inhibits the proliferation and migration of bone tumor cells through the parallel pathways snail1-microRNA181c-NLK and snail1-microRNA181d-TIMP3 [17]. Moreover, Mg^2+^ may alleviate the side effects caused by platinum-based chemotherapeutic agents via the transient receptor potential melastatin 7 channel [18].

Magnesium chloride (MgCl_2_) is an inorganic compound consisting of one magnesium and two chloride ions. It has high water solubility, low toxicity, and is used as a source of Mg^2+^ in medicine, which is essential for many cellular activities. In previous in vitro studies, MgCl_2_ was used as an Mg^2+^ supplement to investigate its influence on bacteria, normal osteoblast cells, tumor cells, etc. [19–21]. Here, MgCl_2_ was added to the RPMI-1640 medium to study the efficacy of Mg^2+^ on the viability, apoptosis, and cell cycle of colorectal tumor cells in vitro. Additionally, the effect of Mg^2+^ on tumor tissue was investigated in animal experiments, by establishing a cell-derived xenograft model in nude mice. Magnesium may play an invaluable role in the post-surgical treatment of CRC.

## Experimental Section

### Cell Culture

The human colorectal adenocarcinoma cell line DLD-1 and RKO was purchased from the Cell Bank of the Chinese Academy of Sciences. The RPMI-1640 medium supplemented with 10% fetal bovine serum (FBS, Gibco, USA) was used to culture cells according to the cell culture guidelines. The cells were passaged twice a week using trypsin (Gibco, USA).

### Cell Viability

The number of 3000 DLD-1 or RKO cells was seeded in each well of 96-well plates. After 24 h of attachment, the culture medium was exchanged for a culture medium supplemented with different concentrations (15 mM and 30 mM) of MgCl_2_ (Aladdin, China) powder. Cell viability was tested at 24, 48, and 72 h by CCK8 assay (DOJINDO, Japan). About 100 μL of the medium-CCK8 solution was added and left for 4 h. The absorbance at 450 nm was then measured by a microplate reader (Spark, Tescan, Czech Republic).

### Live/Dead Staining

After MgCl_2_ addition for 72 h, the DLD-1 cells were conducted live/dead staining. The cells were washed with PBS and stained by calcein-AM (green, live) and ethidiumhomodimer III (red, dead) according to the live/dead staining kit (Biotium, USA). Then, the plates were placed into a cell incubator for 20 min. Finally, the cells were washed once and photographed.

### Cell Apoptosis and Cell Cycle Analysis

Two groups were set up (Mg-supplemented group and normal medium group) to study the influence of Mg on tumor apoptosis. After 72 h of culture, the DLD-1 and RKO cells were harvested by 0.25% trypsin without ethylenediaminetetraacetic acid. After being washed twice with PBS solution, the cells were stained by FITC annexin V and propidium iodide solution from the apoptosis detection kit (Sony Biotechnology, Japan). Finally, the levels of apoptotic cells were tested by a flow cytometer (Beckman Coulter, USA).

The DLD-1 cells were cultured for 72 h in either the RPMI-1640 culture medium with MgCl_2_ or normal RPMI-1640 medium without MgCl_2_. The cells were washed twice with PBS and fixed in 75% ethanol overnight in a − 20 °C fridge. After being stained by a cell cycle kit (BD biosciences, USA), the cell cycle distribution was tested.

### The Immunofluorescence Image of Cleaved Caspase 3

After Mg^2+^ treatment for 72 h, DLD-1 tumor cells were fixed and washed twice. Then, the cells were permeabilized and blocked for 1 h. Subsequently, the cells were stained overnight by the cleaved caspase-3 antibody (1:200, Abcam, UK) and counterstained with a goat anti-rabbit IgG (H&L) secondary antibody (Alexa Fluor® 488, 1:500, Abcam). Confocal laser scanning microscopy (Leica, Germany) was used to capture immunofluorescence images.

### The Effect of Mg on Tumor Model in Vivo

The animal study was approved by the Approval of the Medical Research Ethics Review Committee of General Hospital of Ningxia Medical University. The cell line-derived xenograft (CDX) model was used to construct tumors in BALB/c nude mice (8 weeks, male). The initial concentration of magnesium ions in the serum of BALB/c nude mice was about 2.5 mg% [6]. In brief, 2 × 10^6^ DLD-1 cells were subcutaneously injected into the back of the mice. When the size of the tumor grew over 125 mm^3^, 10 mice were randomly assigned to the Mg injection group (namely Mg group) and control group. The tumor-bearing mice in the control group were injected with 10 μL saline. The Mg group got a 500 mM MgCl_2_ injection every 3 days. On the last day, all mice were sacrificed and the tumor tissue, normal organs were collected and stained by H&E. The tumor weight was recorded and the volume (V) of the tumor tissue was calculated. The tumor tissues from each group were harvested for H&E staining, Ki67 immunohistochemical analysis and transmission electron microscope observation were performed.

### Statistical Analysis

All the data were presented as mean ± SD, and statistical analysis between multiple groups was performed by the one-way ANOVA analysis of variance using GraphPad Prism 8 software (GraphPad, USA). All the figures shown in this article were obtained from experiments that were independently repeated at least three times. Statistical significance is represented by *p < 0.05, **p < 0.01, and ***p < 0.001.

## Results and Discussion

### The Cell Viability

**Fig. 1:**
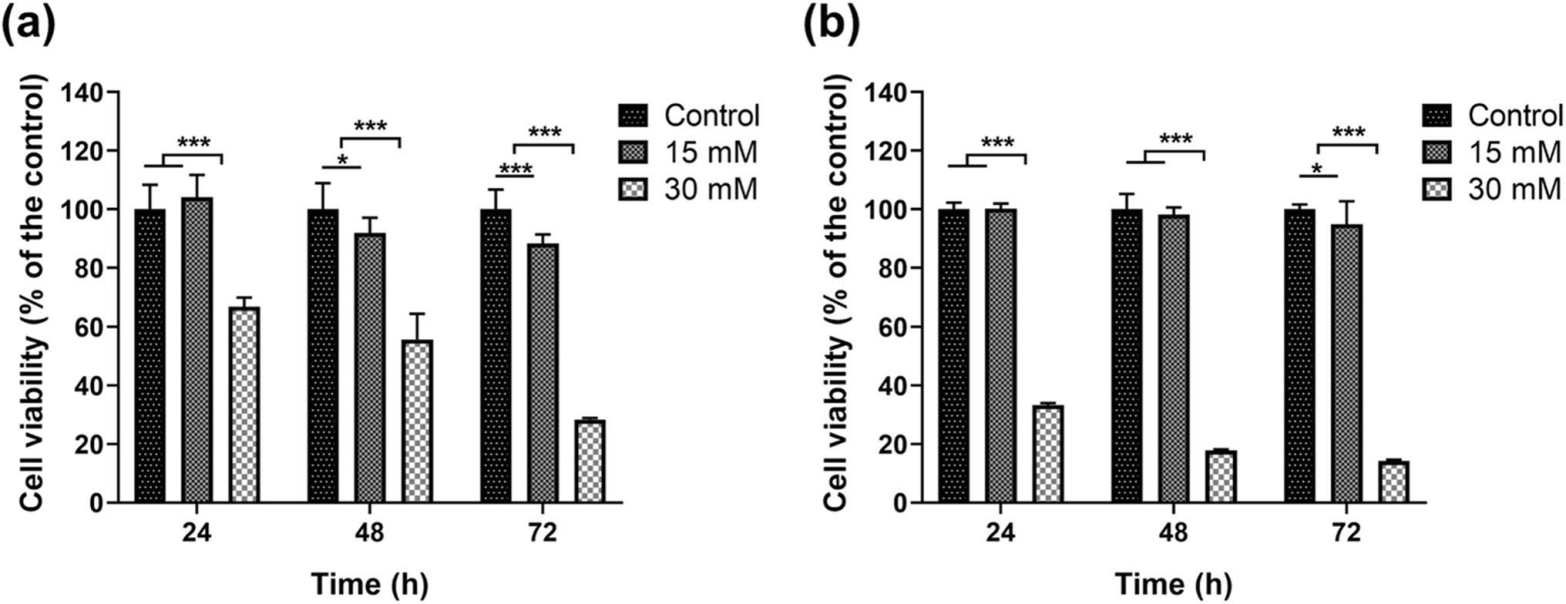
The effect of Mg^2+^ on the viability of DLD-1 (**a**) and RKO (**b**) cells after 24, 48, and 72 h of exposure, respectively. *n* = 12, *p < 0.05, **p < 0.01, ***p < 0.001

To investigate the effect of Mg^2+^ on the viability of colorectal adenocarcinoma cells, the culture medium was supplemented with 15 mM or 30 mM of MgCl_2_. Meanwhile, the culture medium without Mg^2+^ addition was set as the control group. The Mg^2+^ concentration was kept within the normal osmolality range of the cells so that no additional osmotic pressure was exerted [17, 22]. As shown in Fig. 1a, the cell viability of the DLD-1 cells was significantly inhibited after 48 h of Mg^2+^ treatment and showed a gradual decrease as the Mg^2+^ concentration was increased. After 72 h treatment, the cell viability decreased by 71.7% in the 30 mM Mg^2+^ group and by 11.7% in the 15 mM Mg^2+^ group compared to the control group. The RKO cells showed less sensitivity to Mg^2+^ at the 15 mM concentration. After 72-h treatment, the cell viability decreased by 85.8% in the 30 mM Mg^2+^ group and by 5.1% in the 15 mM Mg^2+^ group compared to the control group (Fig. 1b).

**Fig. 2:**
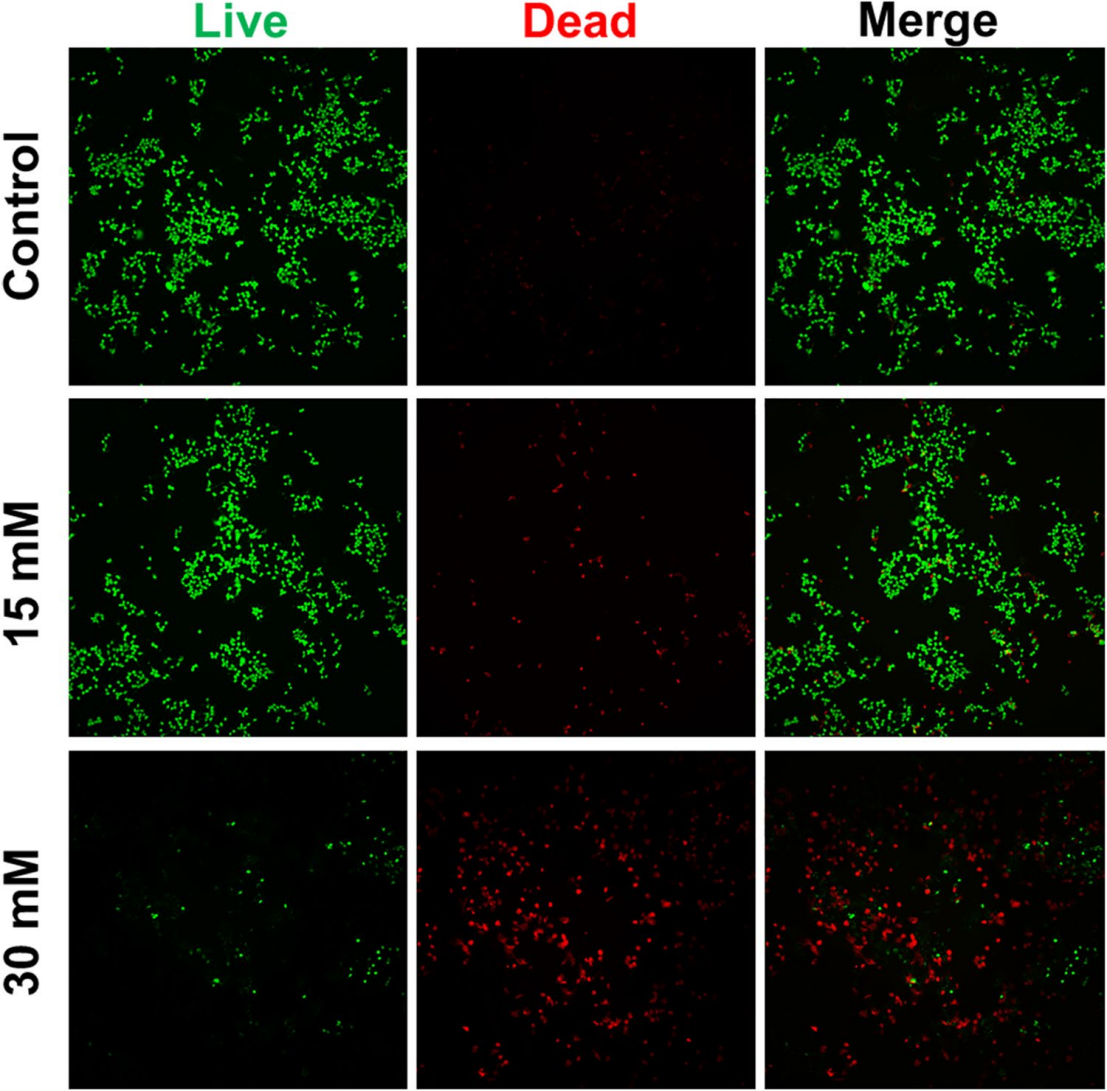
The live/dead staining of DLD-1 cells with different concentrations of Mg^2+^ treatments for 72 h

The live/dead staining also showed that Mg^2+^ suppressed the viability of DLD-1 cells in a dose-dependent manner. As shown in Fig. 2, a high proportion of dead cells (red) to live cells (green) are found in the group supplemented with 30 mM Mg^2+^, while more live cells and fewer dead cells were found in the control group. In addition, the supplement of 15 mM Mg^2+^ increases cell death by a small amount compared to the control group, which is in accordance with the results of Fig. 1.

### Apoptosis Analysis

**Fig. 3:**
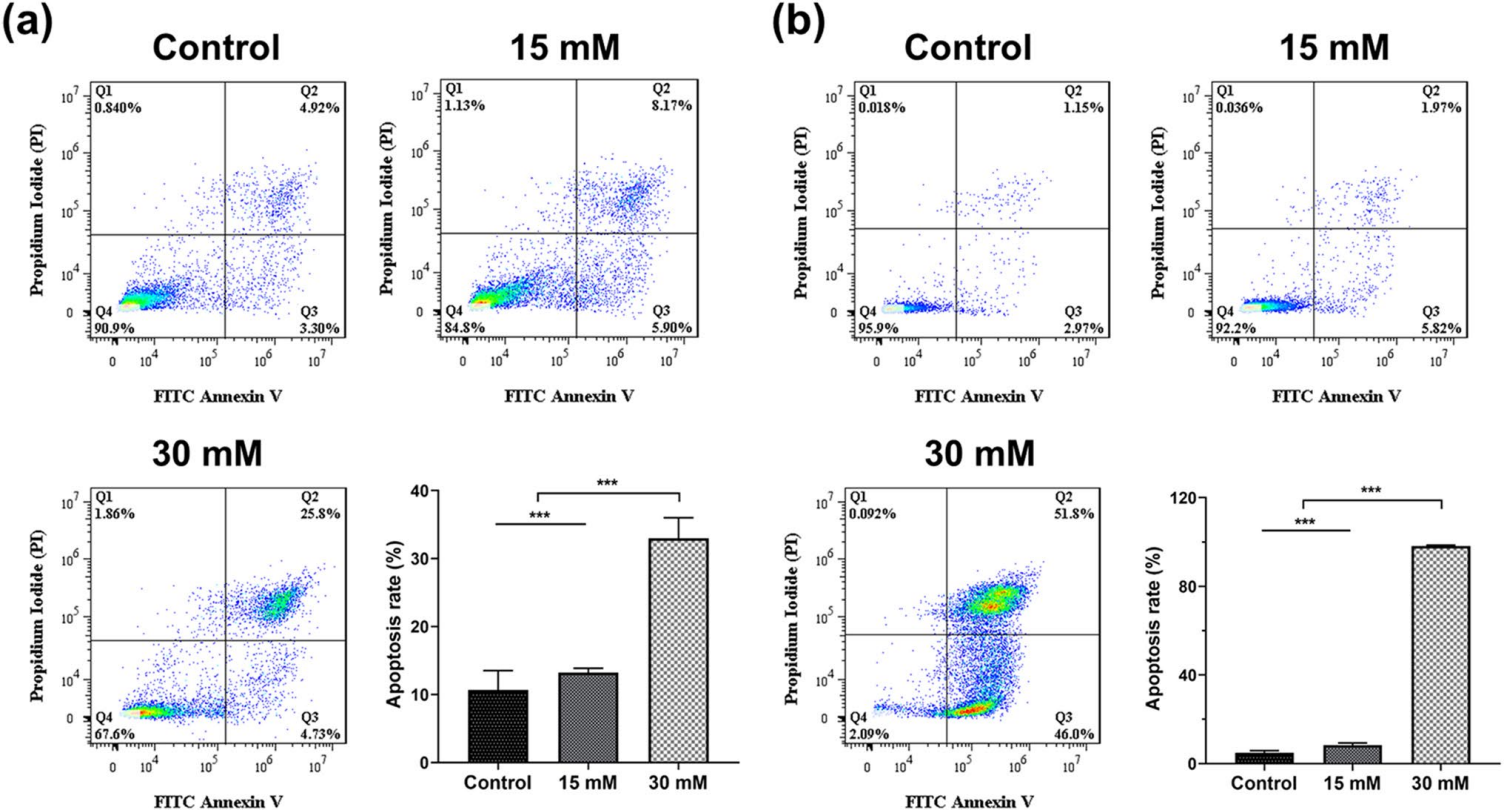
The apoptosis rate and corresponding statistics of DLD-1 (a) and RKO (b) cells with different treatments after 72 h. *n* = 4

Apoptosis is a programmed cell death process, which is an essential factor in clinical oncological therapy to eliminate the tumor cells [23, 24]. In recent studies, compelling evidence has indicated that the initiation of apoptosis effectively inhibits tumor cell growth and subsequently influences proliferation and differentiation [25]. Therefore, the interaction between Mg^2+^ and the incidence of apoptosis in DLD-1 cells and RKO cells was investigated. After 72 h of treatment, the apoptosis rate of DLD-1 cells was proportionally increased with the dose of Mg^2+^, yielding apoptosis rates of 13.2 ± 0.6% and 33.0 ± 3.0% in the 15 mM and 30 mM groups, respectively, which were significantly greater than the control group (10.7 ± 2.8%) (Fig. 3a). Meanwhile, the apoptosis rate of RKO cells in the 15 mM Mg^2+^ medium was 8.4 ± 0.9% and 98.2 ± 0.5% in the 30 mM medium, respectively (Fig. 3b). Mg^2+^ exhibited encouraging apoptotic efficacy on RKO cells.

**Fig. 4:**
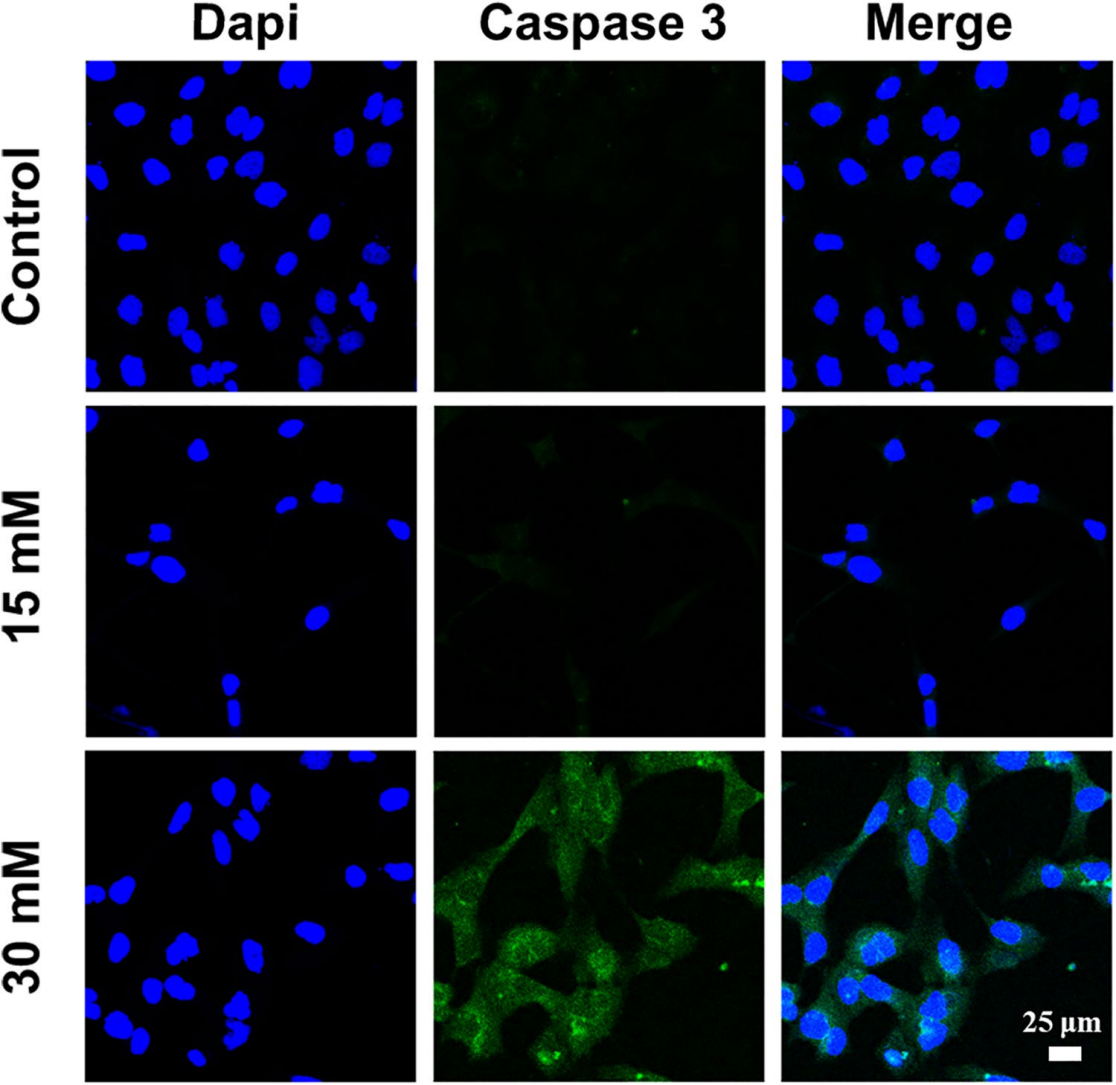
The expression of cleaved caspase 3 of DLD-1 cells for 72 h treatment

The caspase family, especially caspase-3, holds a critical function in programmed cell death and activated protease in apoptosis [26]. FITC-conjugated cleaved caspase-3 was used to evaluate the expression in DLD-1 cells after Mg^2+^ therapy. Among all the groups, the expression of cleaved caspase-3 (green) was the highest in the 30 mM Mg^2+^ group (Fig. 4), indicating increased pro-apoptotic protein levels [27]. The results of the corresponding apoptosis assay and caspase-3 expression jointly indicated that Mg^2+^ could induce apoptosis in colorectal adenocarcinoma DLD-1 cells in vitro.

### The Cell Cycle

**Fig. 5:**
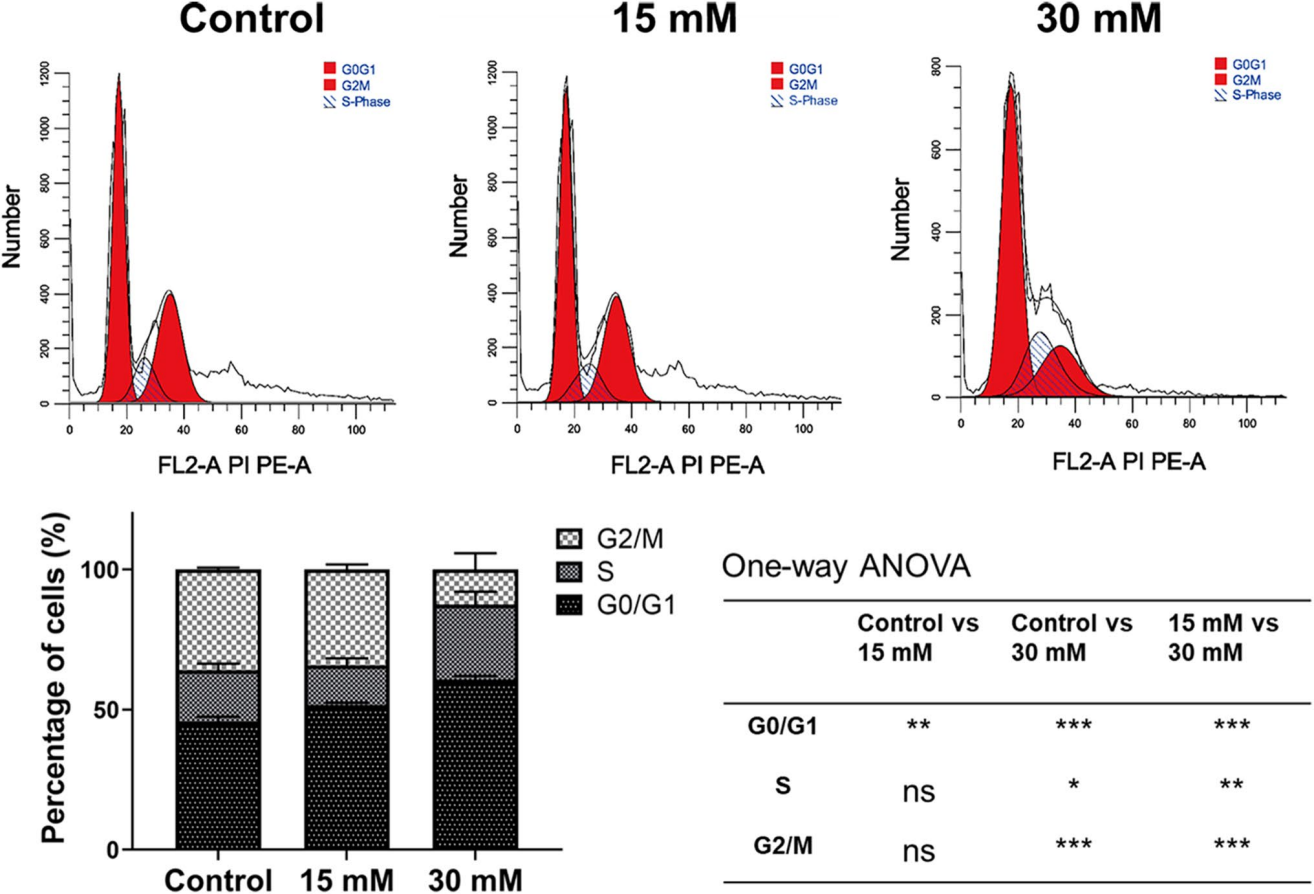
The cell cycle of DLD-1 cells in different groups after treatment with 0, 15, and 30 mM Mg.^2+^ for 72 h. *n* = 4

The link between proliferation and apoptosis is regulated by cell cycle proteins, such as p21, and cdk2 [28–30]. It has been reported that anti-tumor agents inhibit the rapid duplication of tumor cells and arrest tumor cells in the G0/G1 phase to induce apoptosis [31]. This study found that the addition of Mg^2+^ might induce DLD-1 cell arrest in G0/G1 phase. The percentage of cells in the G0/G1 phase in the control group, 15 mM Mg^2+^ group, and 30 mM Mg^2+^ group were 45.7 ± 1.7%, 51.6 ± 1.8%, and 60.6 ± 1.4%, respectively (Fig. 5). Compared to the control group, both the Mg^2+^ supplemented groups showed a significant increase in the percentage of cells arrested in the G0/G1 phase (*p* ˂ 0.001). This finding reveals that Mg^2+^ inhibits proliferation and promotes the apoptosis of colorectal adenocarcinoma cells by inducing cell cycle arrest at the G0/G1 phase [32].

Based on the above results, Mg^2+^ supplementation may promote apoptosis in a dose-dependent manner through regulating the cell cycle of the DLD-1 tumor cells, subsequently inhibiting the proliferation in vitro.

### The Effect of Mg on the Tumor Model in Mice

**Fig. 6:**
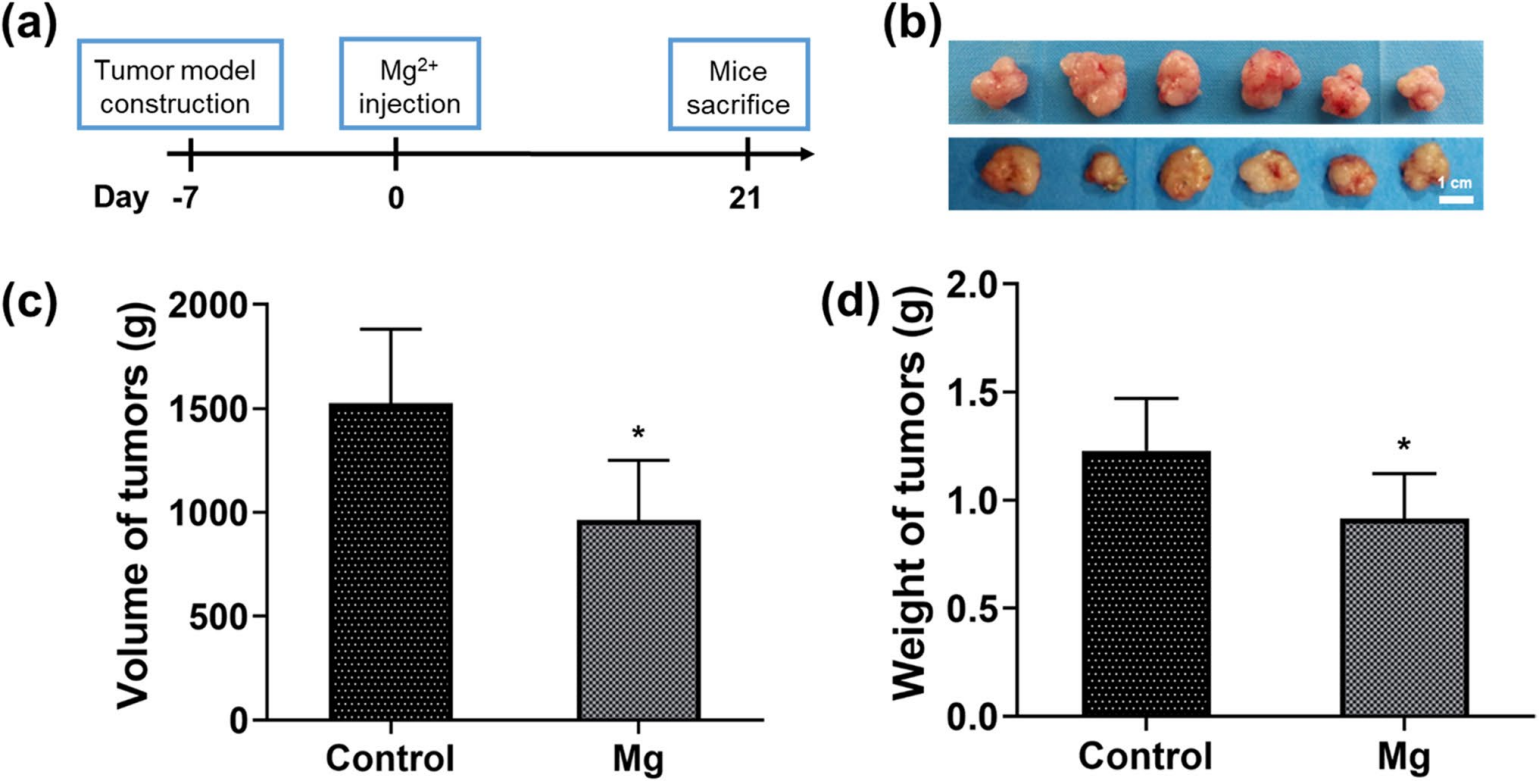
(**a**) Schematic diagram of the Mg ion treatment protocol for tumor therapy in vivo. (**b**) The photograph of the isolated tumor tissue after 21 days of treatment. Scale bar: 1 mm. (**c**, **d**) The volume (**c**) and weight (**d**) of tumors in different groups after 21 days of treatment. *n* = 6

To evaluate the therapeutic effect of Mg^2+^ in vivo, a subcutaneous tumor model was established in BALB/c nude mice. Furthermore, the therapeutic effects of the Mg^2+^ injection were compared with the control group (Fig. 6a). Furthermore, the dose of Mg^2+^ injection was based on previous studies [33]. After treatment for 21 days, all the tumor tissues of mice are harvested, and the volume and weight are measured. As demonstrated in Fig. 6, the volume and weight of tumor tissue were significantly decreased in the Mg group in contrast to the control group. Hence, the results suggested that sufficient Mg^2+^ could efficiently decrease the growth of the tumor tissue in vivo.

**Fig. 7:**
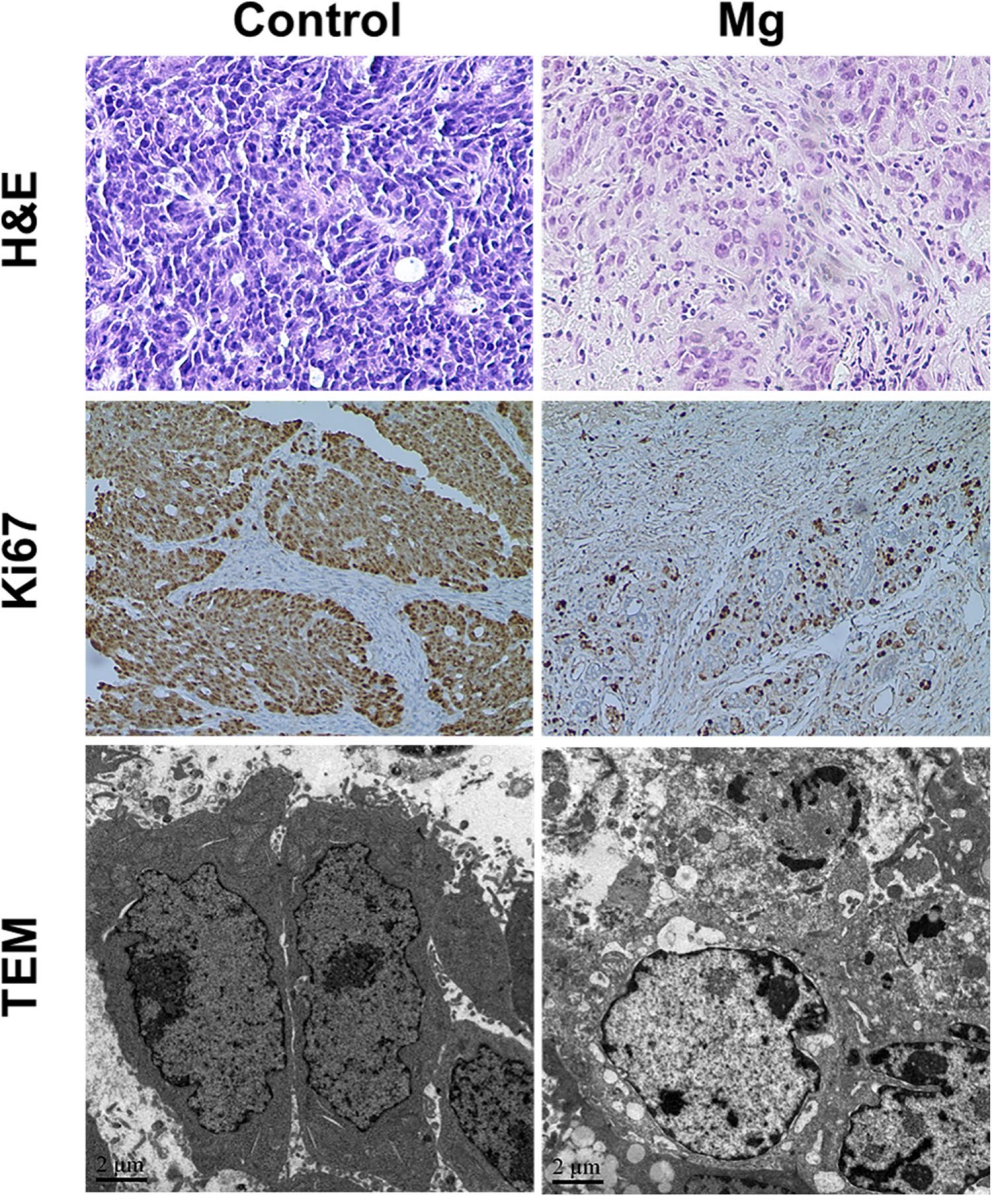
H&E, Ki67, and TEM analysis of the mouse tumor tissues on day 21

As displayed by the H&E staining images, the purple-stained cells indicate that the majority of the tumor cells were alive in the control group. On the contrary, the Mg group induced more tumor apoptosis, resulting in some necrotic areas without abundant inflammatory cell infiltration. In addition, the Ki67 staining was consistent with the H&E staining, showing a higher degree of apoptosis after Mg treatment. Furthermore, examination under the transmission electron microscope (TEM) revealed karyorrhexis and karyolysis of many tumor cell nuclei in the Mg group, which is a typical apoptotic feature (Fig. 7). These results indicated that Mg effectively inhibits the growth and induces apoptosis of colorectal adenocarcinoma DLD-1 cells in vivo, supported by the cell experiments results.

## Conclusion

In summary, this study demonstrated the anti-tumor property of Mg ions in colorectal adenocarcinoma. The Mg ions induce tumor cells apoptosis in a dose-dependent manner through cell cycle G0/G1 arresting, thus inhibiting the proliferation. Intra-articular injection of Mg ions inhibits the growth of tumor tissue in the nude mice. The Mg ions induce tumor apoptosis in the tumor-bearing mice. This study provides additional evidence supporting the use of Mg implants in future surgery.

## Data Availability

The data are available from the corresponding author.

## References

[1] Sung H, Ferlay J, Siegel RL, Laversanne M, Soerjomataram I, Jemal A, Bray F (2021). Global cancer statistics 2020: GLOBOCAN estimates of incidence and mortality worldwide for 36 cancers in 185 countries. CA-Cancer J Clin.

[2] Siegel R, DeSantis C, Jemal A (2014). Colorectal cancer statistics, 2014. CA-Cancer J Clin.

[3] Siegel RL, Miller KD, Goding Sauer A, Fedewa SA, Butterly LF, Anderson JC, Cercek A, Smith RA, Jemal A (2020). Colorectal cancer statistics, 2020. CA-Cancer J Clin.

[4] Chen GC, Pang Z, Liu QF (2012). Magnesium intake and risk of colorectal cancer: a meta-analysis of prospective studies. Eur J Clin Nutr.

[5] Larsson SC, Bergkvist L, Wolk A (2005). Magnesium intake in relation to risk of colorectal cancer in women. JAMA.

[6] Zan R, Wang H, Ni J, Wang W, Peng H, Sun Y, Yang S, Lou J, Kang X, Zhou Y, Chen Y, Yan J, Zhang X (2021). Multifunctional magnesium anastomosis staples for wound closure and inhibition of tumor recurrence and metastasis. ACS Biomater Sci Eng.

[7] Zan R, Wang H, Cai W, Ni J, Luthringer-Feyerabend BJC, Wang W, Peng H, Ji W, Yan J, Xia J, Song Y, Zhang X (2022). Controlled release of hydrogen by implantation of magnesium induces P53-mediated tumor cells apoptosis. Bioact Mater.

[8] Swaminathan R (2003). Magnesium metabolism and its disorders. Clin Biochem Rev.

[9] Ford ES, Mokdad AH (2003). Dietary magnesium intake in a national sample of U.S. adults. J Nutr.

[10] Weglicki WB, Mak IT, Chmielinska JJ, Tejero-Taldo MI, Komarov AM, Kramer JH (2010). The role of magnesium deficiency in cardiovascular and intestinal inflammation. Magnesium Res.

[11] Zhang Y, Xu J, Ruan YC, Yu MK, O'Laughlin M, Wise H, Chen D, Tian L, Shi D, Wang J, Chen S, Feng JQ, Chow DH, Xie X, Zheng L, Huang L, Huang S, Leung K, Lu N, Zhao L, Li H, Zhao D, Guo X, Chan K, Witte F, Chan HC, Zheng Y, Qin L (2016). Implant-derived magnesium induces local neuronal production of CGRP to improve bone-fracture healing in rats. Nat Med.

[12] Zhang K, Lin S, Feng Q, Dong C, Yang Y, Li G, Bian L (2017). Nanocomposite hydrogels stabilized by self-assembled multivalent bisphosphonate-magnesium nanoparticles mediate sustained release of magnesium ion and promote in-situ bone regeneration. Acta Biomater.

[13] Chakraborti S, Chakraborti T, Mandal M, Mandal A, Das S, Ghosh S (2002). Protective role of magnesium in cardiovascular diseases: a review. Mol Cell Biochem.

[14] Serefko A, Szopa A, Wlaź P, Nowak G, Radziwoń-Zaleska M, Skalski M, Poleszak E (2013). Magnesium in depression. Pharmacol Rep.

[15] Yamamoto A, Hiromoto S (2009). Effect of inorganic salts, amino acids and proteins on the degradation of pure magnesium in vitro. Mat Sci Eng C-Mater.

[16] Lötscher J, Martí i Líndez MA-A, Kirchhammer N (2022). Magnesium sensing via LFA-1 regulates CD8+ T cell effector function. Cell.

[17] Zan R, Ji W, Qiao S, Wu H, Wang W, Ji T, Yang B, Zhang S, Luo C, Song Y, Ni J, Zhang X (2021). Biodegradable magnesium implants: a potential scaffold for bone tumor patients. Sci China Mater.

[18] Trapani V, Arduini D, Cittadini A, Wolf FI (2013). From magnesium to magnesium transporters in cancer: TRPM7, a novel signature in tumour development. Magnesium Res.

[19] Robinson DA, Griffith RW, Shechtman D, Evans RB, Conzemius MG (2010). In vitro antibacterial properties of magnesium metal against Escherichia coli, Pseudomonas aeruginosa and Staphylococcus aureus. Acta Biomater.

[20] Wang J, Witte F, Xi T, Zheng Y, Yang K, Yang Y, Zhao D, Meng J, Li Y, Li W, Chan K, Qin L (2015). Recommendation for modifying current cytotoxicity testing standards for biodegradable magnesium-based materials. Acta Biomater.

[21] Zhang Y, Ren L, Li M, Lin X, Zhao H, Yang K (2012). Preliminary study on cytotoxic effect of biodegradation of magnesium on cancer cells. J Mater Sci Technol.

[22] Wu Y, He G, Zhang Y, Liu Y, Li M, Wang X, Li N, Li K, Zheng G, Zheng Y, Yin Q (2016). Unique antitumor property of the Mg-Ca-Sr alloys with addition of Zn. Sci Rep-UK.

[23] Meier P, Finch A, Evan G (2000). Apoptosis in development. Nature.

[24] Carneiro BA, El-Dairy WS (2020). Targeting apoptosis in cancer therapy. Nat Rev Clin Oncol.

[25] Lowe SW, Lin AW (2000). Apoptosis in cancer. Carcinogenesis.

[26] Porter AG, Jänicke RU (1999). Emerging roles of caspase-3 in apoptosis. Cell Death Differ.

[27] Liu C, Liu B, Zhao J, Di Z, Chen D, Gu Z, Li L, Zhao Y (2019). Nd3+-sensitized upconversion metal-organic frameworks for mitochondria-targeted amplified photodynamic therapy. Angew Chem Int Edit.

[28] Alenzi FQB (2004). Links between apoptosis, proliferation and the cell cycle. Br J Biomed Sci.

[29] Gil-Gómez G, Berns A, Brady HJM (1998). A link between cell cycle and cell death: Bax and Bcl-2 modulate Cdk2 activation during thymocyte apoptosis. EMBO J.

[30] Gervais JLM, Seth P, Zhang H (1998). Cleavage of CDK inhibitor p21<sup>Cip1/Waf1</sup> by caspases is an early event during DNA damage-induced apoptosis *. J Biol Chem.

[31] Dothager RS, Putt KS, Allen BJ, Leslie BJ, Nesterenko V, Hergenrother PJ (2005). Synthesis and identification of small molecules that potently induce apoptosis in melanoma cells through G1 Cell Cycle Arrest. J Am Chem Soc.

[32] Qiao S, Wang Y, Zan R, Wu H, Sun Y, Peng H, Zhang R, Song Y, Ni J, Zhang S, Zhang X (2020). Biodegradable Mg implants suppress the growth of ovarian tumor. ACS Biomater Sci Eng.

[33] Yao H, Xu J, Wang J, Zhang Y, Zheng N, Yue J, Mi J, Zheng L, Dai B, Huang W, Yung S, Hu P, Ruan Y, Xue Q, Ho K, Qin L (2021). Combination of magnesium ions and vitamin C alleviates synovitis and osteophyte formation in osteoarthritis of mice. Bioact Mater.

